# Seroprevalence and trends of transfusion-transmissible infections among blood donors in the Volta Region, Ghana: A four-year retrospective study

**DOI:** 10.1371/journal.pone.0349548

**Published:** 2026-10-08

**Authors:** Shadrack Ayanku, Eyram Kuma Hanu, Lois Akuba, Caroline Tetteh, Timothy Yao Akweh, David Annor Kwasie, Stephen Bekpenaneso Bawu, Klutse Fianko, Anthony Zunoo Dongdem

**Affiliations:** 1 Fred Newton Binka School of Public Health, University of Health and Allied Science, Ho, Ghana; 2 Ho Teaching Hospital Blood Bank, Ho, Volta Region, Ghana; 3 Volta Regional Hospital Blood Bank, Hohoe, Volta Region, Ghana; U.S. Food and Drug Administration, UNITED STATES OF AMERICA

## Abstract

**Background:**

Blood transfusion is a life-saving intervention; however, transfusion-transmissible infections (TTIs) such as human immunodeficiency virus (HIV), hepatitis B virus (HBV), hepatitis C virus (HCV), and syphilis remain major public health concerns, particularly in low- and middle-income countries. This study assessed the seroprevalence and temporal trends of TTIs among blood donors in the Volta Region of Ghana and to identify the demographic factors associated with seropositivity.

**Methods:**

A retrospective cross-sectional study was conducted using secondary data from blood donors at Ho Teaching Hospital and Hohoe Regional Hospital between January 2020 and December 2023. Data from 6,147 eligible donors were extracted and analyzed using STATA version 17. Descriptive statistics summarized prevalence, while chi-square or Fisher’s exact tests assessed associations. Multivariable logistic regression was used to identify factors associated with TTI seropositivity at a 5% significance level.

**Results:**

A total of 6,147 blood donors were included in the analysis, of whom 95.1% were male. The overall prevalence of transfusion-transmissible infections (TTIs) was 8.1%, with syphilis (3.6%) being the most prevalent infection, followed by HBV (1.8%), HCV (1.8%), and HIV (1.0%). All infections peaked in 2022 before declining in 2023. Increasing age and year of donation were significantly associated with seropositivity for specific TTIs, particularly HBV, HCV, and syphilis. In Hohoe, male donors had significantly lower odds of HCV infection compared with female donors (aOR = 0.13; 95% CI: 0.06–0.28).

**Conclusions:**

Although TTI prevalence was relatively low, temporal increases and age-related disparities highlight the need for strengthened donor screening, targeted recruitment of voluntary donors, and enhanced surveillance strategies to ensure blood safety.

## Introduction

Blood transfusion is a life-saving medical intervention that has been in use for decades. However, the transmission of transfusion-transmissible infections (TTIs) through blood transfusion remains a significant public health concern [1]. Among transfusion-transmissible infections, HIV, hepatitis B virus (HBV), hepatitis C virus (HCV), and syphilis are of particular public health importance because they are routinely screened in donated blood under WHO blood safety recommendations and continue to contribute substantially to transfusion-related morbidity and mortality worldwide [2–4].

Globally, there are approximately 170 million individuals chronically infected with hepatitis C virus (HCV), more than 350 million with hepatitis B virus (HBV), and 38 million human immunodeficiency virus (HIV)-infected people [3].

Across sub-Saharan Africa, the burden of transfusion-transmissible infections among blood donors remains substantial, although prevalence varies considerably between countries and settings. Recent studies have reported TTI prevalence ranging from less than 5% in some settings to over 10% in others, reflecting differences in donor selection practices, screening strategies, and underlying population disease burden [5–7].

In Ghana, transfusion-transmissible infections among blood donors remain a significant public health concern despite ongoing improvements in blood safety systems. Previous studies have reported varying prevalence estimates across different parts of the country. A national multicentre study reported an overall TTI prevalence of 21.0%, with HBV (6.6%), HCV (4.9%), HIV (2.9%), and syphilis (6.8%) constituting the major infections among blood donors [8]. Similarly, a study conducted in the Ashaiman Municipality reported prevalence rates of 4.9% for HBV, 1.5% for HCV, and 4.0% for syphilis [1], while a study in the Akatsi South Municipality of the Volta Region reported an overall TTI prevalence of 8.0%, comprising HIV (3.8%), HBV (3.2%), and HCV (1.0%) [9]. These findings highlight substantial geographical variation in TTI prevalence and underscore the importance of continuous surveillance to guide blood safety interventions. The World Health Organization (WHO) recommends screening all donated blood for TTIs to ensure the safety of the blood supply [4]. Despite these recommendations, the prevalence of TTIs among blood donors in Ghana remains high, with studies reporting varying prevalence rates depending on the region and population studied [1]. This highlights the need for ongoing surveillance, particularly in areas like Hohoe and Ho, to better understand and mitigate the risks associated with TTIs in blood donation. This study therefore aimed to determine the seroprevalence and temporal trends of transfusion-transmissible infections (TTIs) among blood donors in Hohoe and Ho, Volta Region, Ghana, and to identify demographic factors associated with seropositivity.

## Methods

### Study design

A retrospective cross-sectional study design was employed using secondary data from blood bank records. This design was appropriate for estimating the prevalence of TTIs and examining associations between donor characteristics and TTI seropositivity over the study period

### Study site description

The study was conducted at the Ho Teaching Hospital Blood Bank and the Hohoe Regional Hospital Blood Bank, both located in the Volta Region of Ghana. Ho Teaching Hospital is a tertiary referral facility and a major training center for the University of Health and Allied Sciences, serving the southern Volta Region and neighboring areas. Hohoe Regional Hospital is a key referral facility for the northern Volta and Oti Regions. Together, these facilities provide blood transfusion services to a large and diverse catchment population within the region, which had a total population of approximately 1.66 million people according to the 2021 Population and Housing Census conducted by the Ghana Statistical Service. Both facilities provide blood transfusion services to large and diverse populations and maintain functional blood banks that routinely collect blood from voluntary and family replacement donors.

All donated blood units are screened for transfusion-transmissible infections (TTIs), including HIV, hepatitis B virus (HBV), hepatitis C virus (HCV), and syphilis, in accordance with national blood safety guidelines. The study site is depicted as Fig 1.

**Fig 1:**
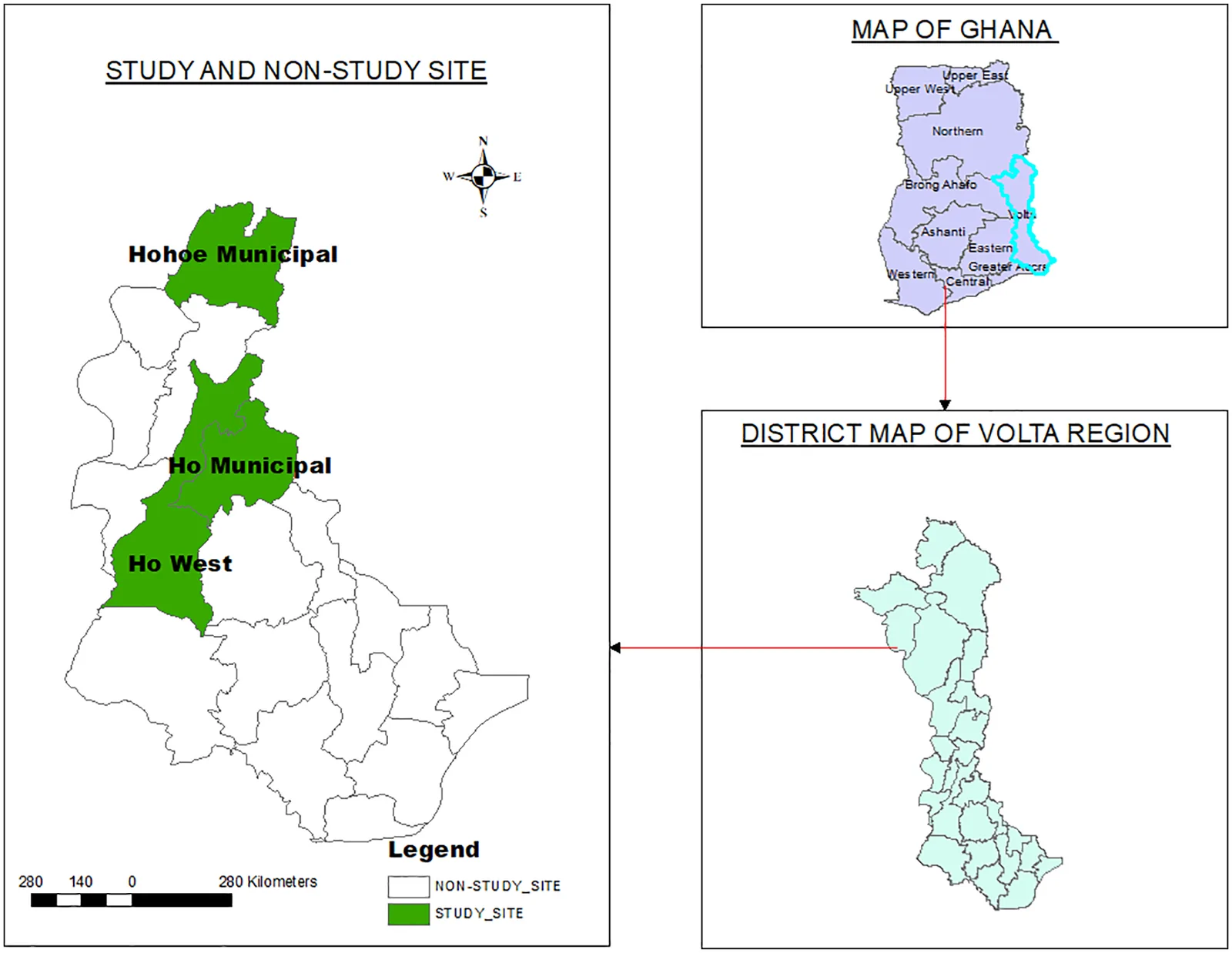
Showing study and non-study site. (Administrative boundary data used in map preparation were obtained from the Ghana Statistical Service and processed using ArcMap).

### Study population

The study population included all individuals who donated blood at the two facilities between January 2020 and December 2023.

### Inclusion criteria

All blood donors whose records contained complete demographic information and TTI screening results for HIV, HBV, HCV, and Syphilis between January 2020 and December 2023 were included in the study.

### Exclusion criteria

Donor records that were incomplete, missing key demographic details, or lacking any of the four TTI test results were excluded from the study.

### Variables

The primary outcome variable was TTI seropositivity (HIV, HBV, HCV, and syphilis). Independent variables included age, sex, and year of donation.

### Sample size determination

All available donor records that met the eligibility criteria during the study period were included. A total of 6,147 donor records were reviewed, comprising 2,015 donors from Ho Teaching Hospital and 4,132 donors from Hohoe Regional Hospital. The inclusion of all eligible records ensured adequate statistical power to estimate prevalence and assess associations.

### Sampling method

Ho Teaching Hospital and Hohoe Regional Hospital were purposively selected because they are major referral facilities with established blood banks and comprehensive donor screening systems. These facilities serve broad catchment populations across the Volta and Oti Regions and maintain detailed records of donor demographics and transfusion-transmissible infection screening results. Their strategic roles in blood collection and transfusion services make them suitable sites for assessing the seroprevalence and trends of transfusion-transmissible infections among blood donors in the region.

### Data collection

Blood donor information was routinely captured using standardized blood bank records maintained at each facility. Donor demographic characteristics, eligibility assessment findings, and laboratory screening results were recorded by trained blood bank personnel at the time of donation. These records formed part of the routine blood bank documentation used for donor management and blood safety monitoring.

Blood donors were screened using standard blood bank procedures involving donor interview, physical examination, and laboratory testing. During the interview, key information such as age, sex, medical history, previous donations, medication use, recent illnesses, travel history, and risk behaviors is asked. Physical assessment including; weight, blood pressure, pulse rate, temperature, and hemoglobin level are measured. Blood samples are then collected from eligible donors and screened for transfusion-transmissible infections including HIV, Hepatitis B, Hepatitis C, and syphilis using Rapid Diagnostic Tests (RDTs) and ELISA. Only donors tested negative to these pathogens are accepted for donation.

### Laboratory screening procedures

Screening for transfusion-transmissible infections (TTIs) was performed using rapid diagnostic tests (RDTs) routinely employed by the participating blood banks during the study period. HIV screening was conducted using the First Response HIV 1/2 rapid test kit. HBV and HCV screening were performed using validated rapid test kits including Accurate, Hightop, Micropoint, and Wondfo, depending on availability during specific periods. Syphilis screening was conducted using Wondfo, Evancare, and Diaspot rapid test kits.

All reactive screening results were subjected to confirmatory testing using Fortress Diagnostics ELISA assays (Fortress Diagnostics Ltd., United Kingdom) corresponding to the infection being tested. Final serological classification was based on the confirmatory ELISA results.

### Data extraction

Data was extracted from blood bank registers from May to June 2025, using a structured data extraction form developed for the study. To ensure data quality, donor records and laboratory results were reviewed for completeness and consistency before extraction. Extracted data were cross-checked against source records to minimize transcription errors and identify missing or inconsistent entries. Records with incomplete information on key study variables were excluded from the analysis. Variables collected included donor age, sex, year of donation, and serological screening results for HIV, HBV, HCV, and syphilis. To ensure data quality, extracted records were cross-checked for completeness and consistency before analysis. Personal identifiers were not collected. The study dataset did not contain unique donor identifiers that permitted reliable linkage of repeat donations across the study period. Consequently, each donation episode was treated as an independent donation event. This approach is consistent with routine blood bank practice, where every donation undergoes a complete donor assessment and laboratory screening irrespective of previous donation history.

### Statistical analysis

Data was entered into Microsoft Excel and exported to STATA version 17 for analysis. Descriptive statistics (frequencies, percentages, means, and standard deviations) were used to summarize donor characteristics and TTI prevalence. Associations between demographic variables and TTI status were assessed using chi-square or Fisher’s exact tests, as appropriate. Variables that demonstrated statistical significance or epidemiological relevance in the bivariate analysis were entered into multivariable logistic regression models. Adjusted odds ratios (aORs) with 95% confidence intervals were reported as measures of association. Multivariable logistic regression models were fitted to identify independent predictors of TTI seropositivity, and adjusted odds ratios (aORs) with 95% confidence intervals (CIs) were reported. Statistical significance was set at p < 0.05.

### Ethics statement

Ethical approval for this study was obtained from the University of Health and Allied Sciences Research Ethics Committee (UHAS-REC), Ghana (Approval number: UHAS-REC B.10 [52]24-25). Permission to access the data was also obtained from the respective health facilities.

The requirement for informed consent was waived by the ethics committee because the study involved secondary analysis of anonymized data with no direct participant contact. All data were handled confidentially and without personal identifiers.

This study was conducted in accordance with the principles of the Declaration of Helsinki.

## Results

### Demographic characteristics

A total of 6,147 blood donors were included in the analysis, comprising 2,015 (32.8%) from Ho and 4,132 (67.2%) from Hohoe. The overall mean age of donors was 26.9 ± 7.4 years, with donors in Ho being slightly older (28.2 ± 7.9 years) than those in Hohoe (26.3 ± 7.1 years). Majority of donors were aged 20–29 years (55.6%), followed by 30–39 years (23.5%). Donors aged ≥50 years constitute less than 1% of the study population. The donor population was predominantly male (95.1%), with females acc ounting for only 4.9% (Tables 1 and 2).

**Table 1:** Demographic characteristics of the blood donors.

Variable	Place		
	Ho n (%)	Hohoe n (%)	
**Mean age (SD)** **Age group**	28.2 (±7.9)	26.3 (±7.1)	26.9 (±7.4)
< 20	187 (9.3)	624 (15.1)	811 (13.2)
20-29	1069 (53.1)	2351 (56.9)	3420 (55.6)
30-39	546 (27.1)	901 (21.8)	1447 (23.5)
40-49	182 (9.0)	236 (5.7)	418 (6.8)
50-59	31 (1.5)	20 (0.5)	51 (0.8)
**Sex**			
Female	133 (6.6)	171 (4.1)	304 (4.9)
Male	1882 (93.4)	3961 (95.9)	5843 (95.1)

**Table 2:** Prevalence of transfusion-transmissible infections among blood donors in the blood banks.

Study site	Year	Total screened	HIV n (%)	HBV n (%)	HCV n (%)	Syphilis n (%)	Overall TTI n (%)
Ho Teaching Hospital	2020	544	11 (2.0)	16 (2.9)	9 (1.7)	9 (1.7)	45 (8.3)
	2021	606	3 (0.5)	10 (1.7)	8 (1.3)	11 (1.8)	32 (5.2)
	2022	437	5 (1.1)	37 (8.5)	17 (3.9)	28 (6.4)	87 (19.9)
	2023	428	8 (1.9)	12 (2.8)	3 (0.7)	14 (3.3)	37 (8.6)
	Total	2015	27 (1.3)	75 (3.7)	37 (1.8)	62 (3.1)	201 (9.9)
Hohoe Regional Hospital	2020	1097	4 (0.4)	4 (0.4)	5 (0.5)	22 (2.0)	35 (3.2)
	2021	1005	1 (0.1)	5 (0.5)	3 (0.3)	26 (2.6)	35 (3.4)
	2022	1022	19 (1.9)	14 (1.4)	39 (3.8)	68 (6.7)	140 (13.7)
	2023	1008	12 (1.2)	13 (1.3)	25 (2.5)	41 (4.1)	91 (9.0)
	Total	4132	36 (0.9)	36 (0.9)	72 (1.7)	157 (3.8)	301 (7.3)
Combined	Overall	6147	63 (1.0)	111 (1.8)	109 (1,8)	219 (3.6)	502 (8.2)

### Overall prevalence of transfusion-transmissible infections

The overall prevalence of transfusion-transmissible infections (TTIs) among blood donors was low. The prevalence of HIV was 1.0% (63/6,147), HBV 1.8% (113/6,147), HCV 1.8% (109/6,147), and syphilis 3.6% (219/6,147). Among the TTIs, syphilis was the most prevalent infection, followed by HBV and HCV, which showed similar prevalence levels.

#### Trends of transfusion-transmissible infections (2020–2023).

Fig 2 shows a clear temporal variation in TTI prevalence over the study period. Across all infections, prevalence was relatively low in 2020 and 2021, followed by a marked increase in 2022, and a subsequent decline in 2023.

**Fig 2:**
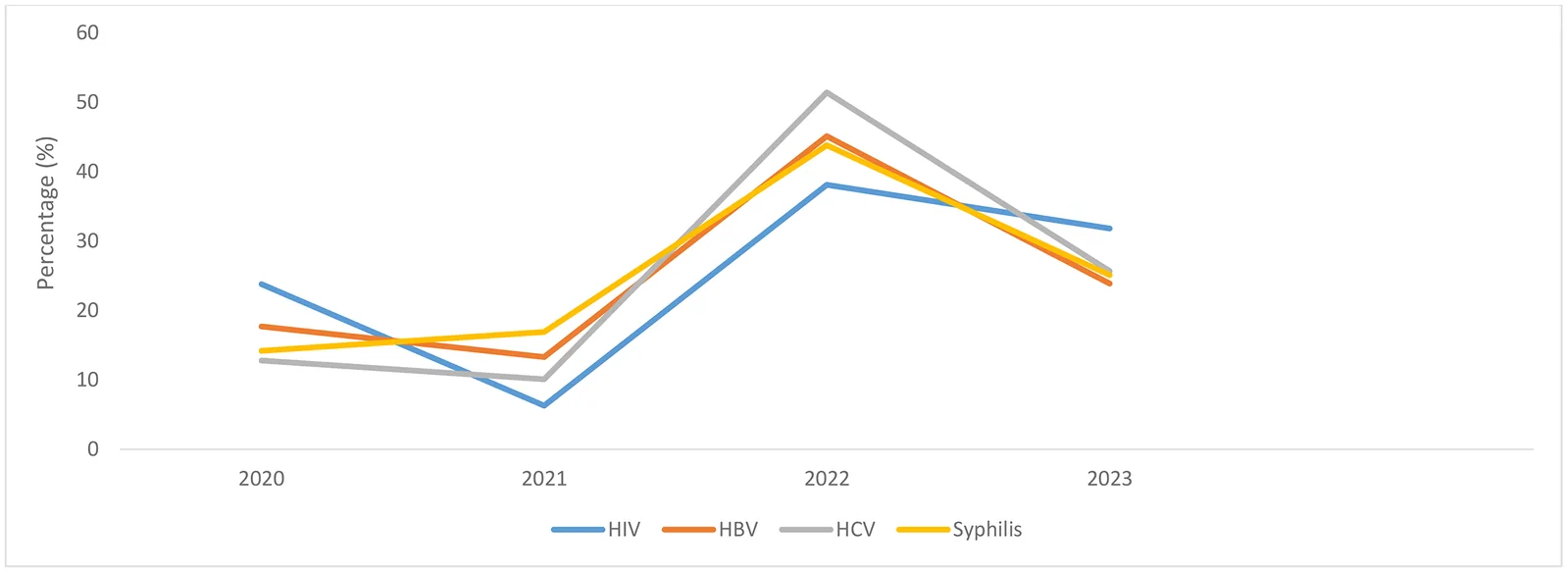
Trends of transfusion-transmissible infections.

Specifically, HIV prevalence increased from 0.3% in 2021 to 1.6% in 2022, while HBV rose from 0.9% to 3.5%, HCV from 0.7% to 3.8%, and syphilis from 2.3% to 6.6% during the same period. Although prevalence declined in 2023, it remained higher than levels observed in 2020–2021 for most infections.

### Site-specific prevalence of TTIs

#### Ho Blood Bank.

Among donors in Ho (n = 2,015), the prevalence of HIV, HBV, HCV, and syphilis was 1.3%, 3.7%, 1.8%, and 3.1%, respectively. HBV was the most prevalent infection in this site. A notable increase in HBV (8.5%) and syphilis (6.4%) was observed in 2022, followed by a decline in 2023 (Table 3).

**Table 3:** Prevalence of transfusion-transmissible infections (TTIs) among blood donors at Ho blood banks.

variable	HIV		Prevalence (%)	HBV		Prevalence (%)	HCV		Prevalence (%)	Syphilis		Prevalence (%)	Total screened
Year	positive	negative		Positive	negative		Positive	negative		Positive	negative		
2020	11	533	2.0	16	528	2.9	9	535	1.7	9	535	1.7	544
2021	3	603	0.5	10	596	1.7	8	598	1.3	11	595	1.8	606
2022	5	432	1.1	37	400	8.5	17	420	3.9	28	409	6.4	437
2023	8	420	1.9	12	416	2.8	3	425	0.7	14	414	3.3	428
Total	27	1988	1.3	75	1940	3.7	37	1978	1.8	62	1953	3.1	2015

#### Hohoe Blood Bank.

Among donors in Hohoe (n = 4,132), the prevalence of HIV, HBV, HCV, and syphilis was 0.9%, 0.9%, 1.7%, and 3.8%, respectively. Syphilis was the most prevalent infection in this site. Similar, to Ho, all infections peaked in 2022, particularly HCV (3.8%) and syphilis (6.7%), with a reduction observed in 2023 (Table 4).

**Table 4:** Prevalence of transfusion-transmissible infections (TTIs) among blood donors at Hohoe blood banks.

variable	HIV		Prevalence (%)	HBV		Prevalence (%)	HCV		Prevalence (%)	Syphilis		Prevalence (%)	Total screened
Year	Positive	Negative		Positive	negative		Positive	negative		Positive	negative		
2020	4	1093	0.4	4	1093	0.4	5	1092	0.5	22	1075	2.0	1097
2021	1	1004	0.1	5	1000	0.5	3	1002	0.3	26	979	2.6	1005
2022	19	1003	1.9	14	1008	1.4	39	983	3.8	68	954	6.7	1022
2023	12	996	1.2	13	995	1.3	25	983	2.5	41	967	4.1	1008
Total	36	4096	0.9	36	4096	0.9	72	4060	1.7	157	3975	3.8	4132

### Factors associated with TTIs: Bivariate analysis

#### Ho Blood Bank.

In Ho, age and year of donation were significantly associated with several TTIs. Increasing age was associated with higher prevalence of HIV (p = 0.02), HBV (p = 0.01), and HCV (p = 0.03). Year of donation was significantly associated with HBV (p < 0.01) and syphilis (p < 0.01), with higher prevalence observed in 2022.

No statistically significant association was observed between gender and any of the TTIs.

#### Hohoe Blood Bank.

In Hohoe, age and year of donation were also significantly associated with TTIs. Age was significantly associated with HBV (p < 0.01), HCV (p = 0.02), and syphilis (p < 0.01), with higher prevalence observed among older donors.

Gender was significantly associated with HCV infection (p < 0.01), with higher prevalence observed among females compared to males. Year of donation were significantly associated with all TTIs (p < 0.01), with peak prevalence consistently observed in 2022 (Tables 5–7).

**Table 5:** Association between demographic characteristics and transfusion-transmissible infections among blood donors in Ho.

Variable	Total n	HIV		HBV		HCV		Syphilis	
**AGE**		**Positive %**	χ**2(p value)**	**Positive %**	χ**2(p value)**	**Positive %**	χ**2(p value)**	**Positive %**	χ**2(p value)**
			0.02*		0.01*		0.03*		0.42*
<20	187	1.1		1.6		0.5		2.7	
20-29	1069	1.1		3.0		1.8		2.6	
30-39	546	1.1		4.6		1.7		3.5	
40-49	182	2.2		7.7		2.8		4.9	
50+	31	9.7		3.2		9.7		3.2	
**GENDER**			0.41*		0.94 *0.33		2.92 *0.08		1.00*
Female	133	2.3		5.3		3.8		3.0	
Male	1882	1.3		3.6		1.7		3.1	
**YEAR**			0.08*		36.64 *p < 0.01		<0.01		23.23 *p < 0.01
2020	544	2.0		2.9		1.7		1.7	
2021	606	0.5		1.7		1.3		1.8	
2022	437	1.1		8.5		3.9		6.4	
2023	428	1.8		2.8		0.7		3.3	

P value is significant if p < 0.05, *Fishers Exact Test

**Table 6:** Association between demographic characteristics and transfusion-transmissible infections among blood donors in Hohoe.

Variable	Total n	HIV		HBV		HCV		Syphilis	
**AGE**		**Positive %**	χ**2(p value)**	**Positive %**	χ**2(p value)**	**Positive %**	χ**2(p value)**	**Positive %**	χ**2(p value)**
			0.17*		<0.01*		0.02*		<0.01*
<20	624	0.6		0.2		1.3		3.0	
20-29	2350	0.8		0.8		1.5		2.9	
30-39	901	0.9		1.3		2.7		5.9	
40-49	236	1.7		1.3		1.7		5.5	
50+	21	4.8		19.1		9.5		19.1	
**GENDER**			1.00*		0.67*		12.91 *P < 0.01		0.41*
Female	171	0.6		1.2		5.3		2.3	
Male	3961	0.9		0.9		1.6		3.9	
**YEAR**			<0.01*		<0.01*		<0.01*		36.67 *P < 0.01
2020	1097	0.4		0.4		0.5		2.0	
2021	1005	0.1		0.5		0.3		2.6	
2022	1022	1.9		1.4		3.8		6.7	
2023	428	1.2		1.5		2.5		4.1	

P value is significant if p < 0.05, *Fishers Exact Test.

**Table 7:** Multivariable logistic regression analysis of factors associated with transfusion-transmissible infections among Ho and Hohoe blood donors.

Variable	HIV, aOR (95% CI)	HBV, aOR (95% CI)	HCV, aOR (95% CI)	Syphilis, aOR (95% CI)
**Ho**				
**Age**				
< 20	Ref	Ref	Ref	Ref
20-29	1.01 (0.22–4.65)	1.72 (0.51–5.72)	3.31 (0.43–25.29)	0.85 (0.32–2.26)
30-39	0.96 (0.18–4.97)	2.55 (0.75–8.67)	3.00 (0.36–24.70)	1.09 (0.40–2.96)
40-49	2.03 (0.36–11.19)	4.36 (1.21–15.61)*	5.24 (0.58–47.16)	1.49 (0.48–4.61)
≥ 50	8.67 (1.37–54.56)*	1.68 (0.17–16.36)	21.66 (2.03–230.75)*	0.92 (0.10–8.00)
**Gender**				
Female	Ref	Ref	Ref	Ref
Male	0.57 (0.17–1.91)	0.68 (0.30–1.52)	0.43 (0.16–1.13)	1.03 (0.35–3.04)
**Year**				
2020	Ref	Ref	Ref	Ref
2021	0.26 (0.07–0.93)*	0.57 (0.25–1.28)	0.89 (0.35–2.27)	1.10 (0.44–2.74)
2022	0.54 (0.18–1.55)	2.90 (1.58–5.34)*	2.35 (1.03–5.35)*	3.99 (1.85–8.60)*
2023	0.87 (0.36–2.11)	0.91 (0.43–1.96)	0.40 (0.11–1.36)	1.98 (0.84–4.67)
**Hohoe**				
**Age**				
< 20	Ref	Ref	Ref	Ref
20-29	1.13 (0.38–3.39)	4.50 (0.59–34.21)	0.94 (0.43–2.07)	0.89 (0.53–1.50)
30-39	1.15 (0.34–3.87)	7.29 (0.93–56.64)	1.57 (0.70–3.52)	1.81 (1.05–3.10)*
40-49	2.38 (0.59–9.58)	7.49 (0.76–73.17)	1.16 (0.34–3.96)	1.77 (0.85–3.67)
≥ 50	10.53 (1.00–110.68)*	82.16 (7.41–910.79)*	13.14 (2.38–72.42)*	9.40 (2.58–34.15)*
**Gender**				
Female	Ref	Ref	Ref	Ref
Male	0.82 (0.11–6.03)	0.49 (0.11–2.09)	0.13 (0.06–0.28)*	1.20 (0.43–3.33)
**Year**				
2020	Ref	Ref	Ref	Ref
2021	0.27 (0.02–2.56)	1.40 (0.36–5.42)	0.70 (0.16–2.95)	1.29 (0.72–2.29)
2022	5.43 (1.83–16.09)*	4.06 (1.26–11.48)*	11.84 (4.56–30.75)*	3.46 (2.12–5.63)*
2023	3.33 (1.07–10.34)*	3.60 (1.12–11.48)*	6.82 (2.54–18.30)*	1.96 (1.16–3.30)*

#### Ho Blood Bank.

After adjusting for potential confounders, age and year of donation remained significant predictors of TTI positivity.

Donors aged ≥50 years had significantly higher odds of HIV infection (aOR = 8.67; 95% CI: 1.37–54.56) and HCV infection (aOR = 21.66; 95% CI: 2.03–230.75) compared to donors aged <20 years.

Donors aged 40–49 years also had increased odds of HBV infection (aOR = 4.36; 95% CI: 1.21–15.61). The year of donation was significantly associated with the incidence of the infections. Compared to 2020, donors in 2022 had higher odds of HBV (aOR = 2.90; 95% CI: 1.58–5.34), HCV (aOR = 2.35; 95% CI: 1.03–5.35), and syphilis (aOR = 3.99; 95% CI: 1.85–8.60).

Gender was not significantly associated with any TTI after adjustment.

#### Hohoe Blood Bank.

In Hohoe, age, gender, and year of donation were significant predictors of TTIs.

Donors aged ≥50 years had significantly higher odds of HBV (aOR = 82.16; 95% CI: 7.41–910.79), HCV (aOR = 13.14; 95% CI: 2.38–72.42), and syphilis (aOR = 9.40; 95% CI: 2.58–34.15) compared to donors aged <20 years.

Male donors had significantly lower odds of HCV infection compared to females (aOR = 0.13; 95% CI: 0.06–0.28).

Year of donation were strongly associated with all TTIs. Compared to 2020, donors in 2022 had significantly higher odds of HIV (aOR = 5.43; 95% CI: 1.83–16.09), HBV (aOR = 4.06; 95% CI: 1.26–11.48), HCV (aOR = 11.84; 95% CI: 4.56–30.75), and syphilis (aOR = 3.46; 95% CI: 2.12–5.63). Elevated odds persisted in 2023 for all infections

## Discussion

This study assessed the seroprevalence and temporal trends of transfusion-transmissible infections (TTIs) among 6,147 blood donors in Ho and Hohoe over a four-year period. The overall TTI prevalence was low with syphilis (3.6%) as the most common infection, followed by HBV (1.8%), HCV (1.8%), and HIV (1.0%). This pattern suggest improved blood safety compared with earlier Ghanaian reports but indicates persistent residual transfusion risk, particularly for vulnerable recipients [2,10].

The observed prevalence is substantially lower than earlier multicenter Ghanaian estimates reporting overall TTI burdens up to 21% and Hohoe-specific historical prevalence exceeding 16% [2,8]. Similar declining trends have been reported in other Ghanaian settings, including Akatsi South (≈8%), suggesting improved donor screening and selection practices across the Volta Region [9]. The reduction likely reflects strengthened pre-donation screening, improved laboratory diagnostics, and stricter donor eligibility criteria implemented over recent years [11,12].

Regionally, findings align with variable, but generally higher TTI burdens have been reported across sub-Saharan Africa. In Nigeria, pooled prevalence estimates are approximately 10%, with HBV typically dominant [10]. In Gabon, HBV (6%), HCV (4%), HIV (3%), and syphilis (3%) remain higher than in the present study [6]. Similarly, Uganda reports an overall prevalence of about 8.7%, with syphilis frequently the leading infection [13]. Southern African pooled estimates remain lower (~2%) but exhibit marked heterogeneity across settings [7].

Globally, high-income countries report TTI prevalence below 1%, largely due to universal voluntary donation systems and advanced screening technologies such as nucleic acid testing (NAT), which significantly reduce window-period infections [14,15]. In contrast, many low- and middle-income countries continue to rely on rapid diagnostic tests, which are less sensitive, potentially underestimating viral infections or missing early-stage disease [16].

The predominance of syphilis in this study contrasts with findings from many West African settings where HBV is often reported as the leading transfusion-transmissible infection but aligns with reports from parts of Ghana, Uganda, and Ethiopia where syphilis remains an important contributor to the burden of TTIs among blood donors [13,17]. The relatively higher prevalence of syphilis may reflect differences in access to sexual and reproductive health services, variations in community-level STI screening and treatment programmes, and delayed healthcare-seeking behaviour among infected individuals.

Furthermore, syphilis infection is frequently asymptomatic during certain stages, allowing infected individuals to remain undiagnosed and untreated for prolonged periods. Regional differences in the implementation of STI prevention initiatives, public awareness programmes, and partner notification services may also contribute to variations in syphilis prevalence between settings [18,19]. While our study did not collect behavioural or cultural data to directly evaluate these factors, they represent plausible explanations that warrant further investigation in future studies. Additionally, differences in test sensitivity may contribute to relative pattern shifts. Treponemal rapid tests, while widely used, may yield variability in detection compared with ELISA-based or confirmatory assays, potentially influencing observed prevalence distributions [16]. The persistence of syphilis as the leading infection underscores the need for strengthened community-level prevention strategies and improved diagnostic algorithms.

A notable finding is the peak in all TTIs in 2022, followed by a decline in 2023. This pattern is consistent with broader African and global reports documenting post-COVID-19 disruptions in blood safety systems [20,21]. The increase in TTI prevalence observed in 2022 should also be interpreted with caution. Different rapid diagnostic test brands were used across the study period based on availability within the participating facilities. Although all reactive screening results were confirmed using Fortress Diagnostics ELISA assays, variations in the diagnostic performance characteristics of screening kits may have contributed to some fluctuations in observed prevalence. Nevertheless, the standardized ELISA confirmation process likely minimized substantial differences in final infection classification across years.

During the pandemic, reduced voluntary donations, increased reliance on replacement donors, and strained laboratory capacity likely contributed to temporary increases in TTI prevalence [22,23].

Similar fluctuations have been reported in Ethiopia, Somalia, Cameroon, and Brazil, where health system shocks altered donor recruitment dynamics and screening efficiency [24–26]. The subsequent decline in 2023 likely reflects restoration of routine services, improved donor recruitment strategies, and reactivation of public health interventions [8,12]. These findings highlight the vulnerability of blood safety systems to external shocks and the importance of resilient haemovigilance frameworks.

Age was a consistent predictor of TTI positivity, with older donors showing higher risk, particularly for HBV and HCV. This is consistent with evidence from multiple African and Asian studies, where cumulative exposure increases infection probability over time [17,26,27]. However, some studies also report higher HBV/HCV prevalence in younger donors in high-risk recruitment systems, highlighting context-specific variation [8,15].

Gender differences were more limited but showed a significant association between male sex and HCV positivity in Hohoe. This aligns with studies from Ethiopia and Angola, where males often exhibit higher HBV/HCV prevalence due to greater representation among donors and higher exposure risk behaviors [17,27]. Conversely, some settings report higher female seroprevalence due to antenatal screening detection bias, illustrating heterogeneous gender patterns across regions [10,28]. Donation year was also a significant predictor, reflecting broader system-level influences such as pandemic disruptions, policy changes, and evolving screening technologies [8,20]. Such temporal variability highlights the importance of continuous surveillance in interpreting TTI trends.

A key structural determinant of TTI risk is reliance on replacement or family donors, who consistently demonstrate higher infection rates than voluntary non-remunerated donors [12,28]. This remains a major challenge in many African blood systems and likely contributes to persistent infections despite overall declining trends. Socio-demographic factors such as younger age distribution, male dominance, and lower repeat donation rates further exacerbate residual risk [8,9].

## Conclusion

This study determined a TTI seroprevalence of 8.1% among 6,147 blood donors at two blood banks in Ghana’s Volta Region (2020–2023), which is substantially lower than the national multicentered benchmark of 21.0% and prior Hohoe rates exceeding 16.1% [8,12]. Seroprevalence peaked in 2022 across all pathogens before declining in 2023. Older age was consistently associated with seropositivity, and site-specific burdens of syphilis (3.6%) and HBV/HCV (1.8% each) were identified. The predominance of young male donors and the contribution of replacement donors emerged as key vulnerabilities, particularly during pandemic-related disruptions.

These findings affirm that sustained implementation of national transfusion safety protocols has strengthened the blood supply. However, persistent reservoirs of TTIs necessitate targeted actions: expanding voluntary non-remunerated donor recruitment, integrating point-of-care syphilis testing, adding anti-HBc screening to detect occult HBV infections, and enhancing female and older-donor recruitment. Future prospective multicenter studies incorporating molecular assays and behavioral risk stratification will be essential to further reduce residual risks and ensure Ghana’s blood system meets universal safety standards.

### Strength and limitations

#### Strengths.

This study included all eligible blood donor records over a four-year period, resulting in a large sample size that improves the reliability of the findings. The inclusion of data from two major blood banks in the Volta Region also allowed for comparisons between sites and provides a broader view of transfusion-transmissible infections (TTIs) in the area.

The use of multivariable analysis made it possible to identify key factors associated with TTIs, particularly age and year of donation, while accounting for potential confounding. In addition, the assessment of trends over time provided useful insight into how the prevalence of infections changed across the study period. The use of routinely collected data based on standard screening procedures also makes the findings relevant to real-world blood safety practices.

#### Limitations.

Information on donor occupation was not available in the blood bank records used for this study. Occupational characteristics may influence exposure to transfusion-transmissible infections through differences in work-related risks, mobility patterns, and health-seeking behaviours. The absence of this information limited our ability to assess occupation as a potential factor associated with TTI seropositivity.

The study was unable to estimate the residual risk of transfusion-transmissible infections among blood donors. Estimation of residual risk typically requires incidence data, repeat donor follow-up information, and modelling of diagnostic window periods, which were not available within the routine blood bank records used for this study. Consequently, the study could not quantify the potential risk of infectious donations that may not have been detected through routine screening procedures.

Although reactive screening results were confirmed using ELISA assays, the possibility of diagnostic misclassification cannot be completely excluded. Serological assays may occasionally yield false-positive or false-negative results due to factors such as cross-reactivity, biological variability, specimen handling conditions, or inherent test performance characteristics. Consequently, the reported prevalence estimates should be interpreted with this limitation in mind.

Although the study included data from two major referral hospitals serving large catchment populations, the findings may not be fully generalizable to all blood donors in the Volta Region, Oti Region, or Ghana. Sociodemographic characteristics, healthcare access, donor recruitment patterns, and infection risk profiles may differ across geographical areas and healthcare facilities, potentially influencing the prevalence and distribution of transfusion-transmissible infections.

The retrospective design limits the ability to establish causal relationships and introduces the possibility of unmeasured confounding factors, such as behavioral risks and donor type, including voluntary or replacement donors. This may have influenced the estimated prevalence of transfusion-transmissible infections, as family or replacement donors may conceal high-risk behaviours.

In addition, the study relied on secondary data obtained from routine screening records. As such, the analysis was dependent on the testing methods originally used at the facilities, and it was not possible to verify or standardize these procedures. This may have led to an underestimation of the true prevalence, particularly if infections occurring during the window period were not detected.

The study population was also skewed, with a predominance of male donors and a relatively young age distribution, while females and older individuals were underrepresented. This limits the generalizability of the findings and reduces the statistical power for subgroup analyses by age and gender, a pattern that is consistent with other studies in the region.

## Supporting information

S1 File TTI data. (XLSX)
